# High level review of configuration and reform of Public Health systems in selected countries

**DOI:** 10.1093/eurpub/ckac129.206

**Published:** 2022-10-25

**Authors:** K Cardwell, N Broderick, B Tyner, G Masukume, P Harrington, M Connolly, L Larkin, B Clyne, M Ryan, M O'Neill

**Affiliations:** 1 Health Technology Assessment, Health Information and Quality Authority, Dublin, Ireland; 2 School of Medicine, National University of Ireland Galway, Galway, Ireland; 3 Department of General Practice, RCSI University of Medicine and Health Sciences, Dublin, Ireland; 4 Department of Pharmacology and Therapeutics, Trinity College Dublin, Dublin, Ireland

## Abstract

**Background:**

The impact of the COVID-19 pandemic has prompted governments internationally to consider reform and strengthening of their Public Health systems. To support this work in Ireland, we undertook a review Public Health systems internationally (research question [RQ] 1), and identified lessons learned from the COVID-19 pandemic (RQ2).

**Methods:**

Data relating to Public Health systems (RQ1), and lessons learned (RQ2) for a select group of 12 countries were identified from organisations’ websites, an electronic database and grey literature search and representatives from key national-level organisations. Data for RQ1 were extracted, mapped to the 12 Essential Public Health functions (EPHFs) at national, regional and local levels, and verified by participating representatives. For RQ2, thematic analysis of semi-structured interviews with participating representatives was undertaken and.

**Results:**

Typically, across all included countries, there is national strategic oversight of all EPHFs and, for certain functions, there is regional and local level implementation. Lessons learned from the COVID-19 pandemic broadly related to the themes of legislation and decision making; data collection, surveillance, evidence synthesis and collaboration; public health interventions; public participation, public messaging and communication; continuation of healthcare services; and workforce capacity and resilience.

**Conclusions:**

When structuring Public Health systems, there is a need to identify which functions, and or which elements of a function, should be delivered at a national, regional or local level to ensure a sustainable and comprehensive Public Health system. Appropriate IT infrastructure, strong communication and an established evidence synthesis function are key to timely and informed decision making. Ideally, these functions should be established during periods of relative stability to permit a faster response during a pandemic or emergency situation.

